# (2*RS*,8a*RS*)-6-Oxo-1,2,3,4,6,7,8,8a-octa­hydro­naphthalene-2-carboxylic acid

**DOI:** 10.1107/S1600536808035691

**Published:** 2008-11-08

**Authors:** Georgia Efthimiopoulos, Roger A. Lalancette, Hugh W. Thompson

**Affiliations:** a Carl A. Olson Memorial Laboratories, Department of Chemistry, Rutgers University, Newark, NJ 07102, USA

## Abstract

The title racemate, C_11_H_14_O_3_, aggregates in the crystal structure as acid-to-ketone O—H⋯O hydrogen-bonding catemers whose components are glide-related. The relative stereochemistry at the carboxyl group arises spontaneously during the synthesis. Two inter­molecular C—H⋯O=C close contacts were found, both involving the acid group.

## Related literature

For background information, see: Borthwick (1980 ▶). For synthetic details see: Finnegan & Bachman (1965 ▶); House *et al.* (1965 ▶). For information on weak hydrogen bonds, see: Steiner (1997 ▶).
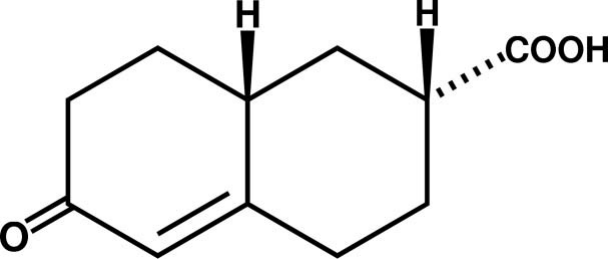


## Experimental

### 

#### Crystal data

C_11_H_14_O_3_
*M* *_r_* = 194.22
Monoclinic, 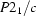

*a* = 6.2315 (11) Å
*b* = 9.2296 (16) Å
*c* = 17.234 (3) Å
β = 93.366 (3)°
*V* = 989.5 (3) Å^3^
*Z* = 4
Cu *K*α radiation
μ = 0.77 mm^−1^
*T* = 100 (2) K
0.36 × 0.31 × 0.22 mm

#### Data collection

Bruker SMART APEXII CCD area-detector diffractometer
Absorption correction: multi-scan (**SADABS**; Sheldrick, 2001 ▶) *T*_min_ = 0.768, *T*_max_ = 0.849
7466 measured reflections
1719 independent reflections
1684 reflections with *I* > 2σ(*I*)
*R*_int_ = 0.028

#### Refinement

*R*[*F* ^2^ > 2σ(*F* ^2^)] = 0.035
*wR*(*F* ^2^) = 0.088
*S* = 1.09
1719 reflections
131 parameters
H atoms treated by a mixture of independent and constrained refinement
Δρ_max_ = 0.22 e Å^−3^
Δρ_min_ = −0.20 e Å^−3^

### 

Data collection: *APEX2* (Bruker, 2006 ▶); cell refinement: *SAINT* (Bruker, 2005 ▶); data reduction: *SAINT*; program(s) used to solve structure: *SHELXTL* (Sheldrick, 2008 ▶); program(s) used to refine structure: *SHELXTL*; molecular graphics: *SHELXTL*; software used to prepare material for publication: *SHELXTL*.

## Supplementary Material

Crystal structure: contains datablocks I, global. DOI: 10.1107/S1600536808035691/lh2720sup1.cif

Structure factors: contains datablocks I. DOI: 10.1107/S1600536808035691/lh2720Isup2.hkl

Additional supplementary materials:  crystallographic information; 3D view; checkCIF report

## Figures and Tables

**Table 1:** Hydrogen-bond geometry (Å, °)

*D*—H⋯*A*	*D*—H	H⋯*A*	*D*⋯*A*	*D*—H⋯*A*
O3—H3⋯O1^i^	0.888 (19)	1.79 (2)	2.6797 (13)	174.8 (17)
C2—H2⋯O2^ii^	1.00	2.40	3.3191 (15)	152
C7—H7*A*⋯O2^iii^	0.99	2.47	3.3708 (15)	151

Symmetry codes: (i) 
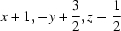
; (ii) 
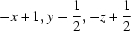
; (iii) 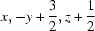.

**Fig. 1.**
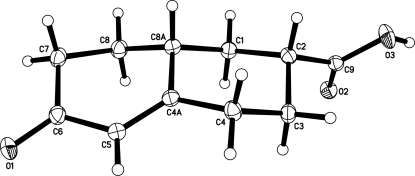
The asymmetric unit of (I), with its numbering. Displacement ellipsoids are drawn at the 40% probability level.

**Fig. 2.**
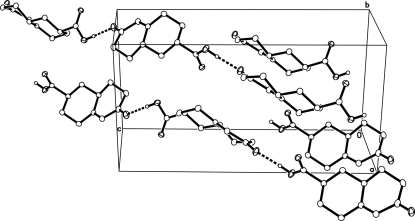
A partial packing diagram with extracellular molecules, illustrating the centrosymmetrically related pairs of acid-to-ketone H-bonding chains passing through the cell. All carbon-bound H atoms are removed for clarity. Displacement ellipsoids are drawn at the 40% probability level.

## supplementary crystallographic information

### Comment

Among ketocarboxylic acids, we have shown that the usually dominant dimerization can be disfavored by lowering molecular flexibility, as measured by the number of fully rotatable bonds present. Typically this results in increased occurrence of acid-to-ketone catemers, whose occurrence is also favored by fixed "anti-like" arrangements, in which carboxyl and ketone are aimed in opposite directions. In this context, we report here the title compound, (I), whose structure conforms to both of the above criteria.

Fig. 1 shows the asymmetric unit, whose only conformational options lie in the carboxyl side-chain, which is oriented [C1—C2—C9—O2 torsion angle = -37.96 (15)°] so as to minimize steric interactions with H atoms at C1 and C3.

The disordering of C—O bond lengths and C—C—O angles often seen in carboxyl dimers becomes impossible when the H-bonding mode precludes the required averaging mechanisms. Because (I) is not dimeric the distances and angles here are fully ordered and thus typical of those in highly ordered dimeric carboxyls (Borthwick, 1980).

Fig. 2 shows the packing of the cell, with extra molecules included to illustrate the acid-to-ketone H-bonding scheme. Each carboxylic acid is linked to the ketone in a molecule glide related in the c direction. Glide relationships for intra-chain units in catemers is far less common than screw-related schemes. Each of the four molecules in the chosen cell participates in a separate H-bonding chain and these pass through the cell in counterdirectional pairs related by centrosymmetry, with the chains advancing by one cell in a and one-half cell in c for each H bond.

We characterize the geometry of H bonding to carbonyls using a combination of the H···O=C angle and the H···O=C—C torsion angle. These describe the approach of the H atom to the receptor O in terms of its deviation from, respectively, C=O axiality (ideal = 120°) and planarity with the carbonyl (ideal = 0°). In (I), these angles are 131.0 (6) & 0.6 (8)°.

Within the 2.6 Å range we standardly survey for C—H···O packing interactions (Steiner, 1997), two intermolecular close contacts were found, both involving O2, the carboxyl carbonyl (see table).

### Experimental

Compound (I) was synthesized by the method of Finnegan & Bachman (1965); crystallization from ethyl acetate yielded material suitable for X-ray, mp 418 K. The C2/C8a stereochemistry clearly represents the stabler of the two epimers possible and probably arises as the result of equilibrations during the synthesis (House *et al.*, 1965).

The solid-state (KBr) infrared spectrum of (I) has C=O absorptions at 1721 & 1640 cm^-1^, with a peak separation typical of the shifts seen in catemers, due, respectively, to removal of H bonding from the acid C=O and addition of H bonding to the ketone; an alkene peak appears at 1616 cm^-1^. In CHCl_3_ solution, where dimers predominate, these bands appear, respectively, at 1708, 1666 and 1622 cm^-1^.

### Refinement

All H atoms for (I) were found in electron-density difference maps. The positional parameters for the carboxyl H were allowed to refine but the *U*_iso_(H) was held at 1.5*U*_eq_(O). The methylene, methine and vinyl Hs were placed in geometrically idealized positions and constrained to ride on their parent C atoms with C–H distances of 0.99, 1.00 & 0.95 Å, respectively, and *U*_iso_(H) = 1.2*U*_eq_(C).

### Crystal data

**Table:**

C_11_H_14_O_3_	*F*_000_ = 416
*M**_r_* = 194.22	*D*_x_ = 1.304 Mg m^−^^3^
Monoclinic, *P*2_1_/*c*	Melting point: 418 K
Hall symbol: -P 2ybc	Cu *K*α radiation λ = 1.54178 Å
*a* = 6.2315 (11) Å	Cell parameters from 7006 reflections
*b* = 9.2296 (16) Å	θ = 4.8–67.1º
*c* = 17.234 (3) Å	µ = 0.77 mm^−^^1^
β = 93.366 (3)º	*T* = 100 (2) K
*V* = 989.5 (3) Å^3^	Parallelepiped, colourless
*Z* = 4	0.36 × 0.31 × 0.22 mm

### Data collection

**Table:**

Bruker SMART CCD APEXII area-detector diffractometer	1719 independent reflections
Radiation source: fine-focus sealed tube	1684 reflections with *I* > 2σ(*I*)
Monochromator: graphite	*R*_int_ = 0.028
*T* = 100(2) K	θ_max_ = 67.3º
φ and ω scans	θ_min_ = 5.1º
Absorption correction: multi-scan(*SADABS*; Sheldrick, 2001)	*h* = −7→7
*T*_min_ = 0.769, *T*_max_ = 0.849	*k* = −11→10
7466 measured reflections	*l* = −20→20

### Refinement

**Table:**

Refinement on *F*^2^	Hydrogen site location: inferred from neighbouring sites
Least-squares matrix: full	H atoms treated by a mixture of independent and constrained refinement
*R*[*F*^2^ > 2σ(*F*^2^)] = 0.035	*w* = 1/[σ^2^(*F*_o_^2^) + (0.0417*P*)^2^ + 0.4167*P*] where *P* = (*F*_o_^2^ + 2*F*_c_^2^)/3
*wR*(*F*^2^) = 0.088	(Δ/σ)_max_ < 0.001
*S* = 1.09	Δρ_max_ = 0.22 e Å^−^^3^
1719 reflections	Δρ_min_ = −0.20 e Å^−^^3^
131 parameters	Extinction correction: *SHELXTL* (Sheldrick, 2004), Fc^*^=kFc[1+0.001xFc^2^λ^3^/sin(2θ)]^-1/4^
Primary atom site location: structure-invariant direct methods	Extinction coefficient: 0.0064 (8)
Secondary atom site location: difference Fourier map	

### Special details

**Table:**

Experimental. crystal mounted on a Cryoloop using Paratone-N
Geometry. All e.s.d.'s (except for the dihedral angle between two l.s. planes) are estimated using the full covariance matrix. The cell e.s.d.'s are taken into account individually in the estimation of e.s.d.'s in distances, angles and torsion angles; correlations between e.s.d.'s in cell parameters are only used when defined by crystal symmetry. An approximate (isotropic) treatment of cell e.s.d.'s is used for estimating e.s.d.'s involving l.s. planes.
Refinement. Refinement of *F*^2^ against ALL reflections. The weighted *R*-factor *wR* and goodness of fit *S* are based on *F*^2^, conventional *R*-factors *R* are based on *F*, with *F* set to zero for negative *F*^2^. The threshold expression of *F*^2^ > σ(*F*^2^) is used only for calculating *R*-factors(gt) *etc*. and is not relevant to the choice of reflections for refinement. *R*-factors based on *F*^2^ are statistically about twice as large as those based on *F*, and *R*- factors based on ALL data will be even larger.

### Fractional atomic coordinates and isotropic or equivalent isotropic displacement parameters (Å^2^)

**Table:**

	*x*	*y*	*z*	*U*_iso_*/*U*_eq_	
O1	−0.28896 (14)	0.72032 (11)	0.52340 (5)	0.0292 (3)	
C1	0.36320 (19)	0.76724 (13)	0.32544 (7)	0.0191 (3)	
H1A	0.2697	0.8459	0.3038	0.023*	
H1B	0.5053	0.8100	0.3405	0.023*	
O2	0.41479 (13)	0.84304 (9)	0.16676 (5)	0.0217 (2)	
C2	0.39130 (18)	0.65372 (13)	0.26199 (7)	0.0176 (3)	
H2	0.4969	0.5788	0.2816	0.021*	
O3	0.61028 (15)	0.64301 (10)	0.15353 (5)	0.0281 (3)	
H3	0.652 (3)	0.689 (2)	0.1116 (11)	0.042*	
C3	0.17383 (19)	0.58079 (14)	0.24029 (7)	0.0199 (3)	
H3A	0.0716	0.6537	0.2178	0.024*	
H3B	0.1932	0.5052	0.2006	0.024*	
C4	0.08193 (19)	0.51220 (13)	0.31220 (7)	0.0200 (3)	
H4A	−0.0631	0.4735	0.2978	0.024*	
H4B	0.1744	0.4299	0.3297	0.024*	
C4A	0.06671 (19)	0.61740 (13)	0.37828 (7)	0.0175 (3)	
C5	−0.11075 (19)	0.62502 (14)	0.41883 (7)	0.0198 (3)	
H5	−0.2346	0.5720	0.4009	0.024*	
C6	−0.1206 (2)	0.71159 (14)	0.48942 (7)	0.0210 (3)	
C7	0.0842 (2)	0.78214 (14)	0.52003 (7)	0.0223 (3)	
H7A	0.1681	0.7127	0.5534	0.027*	
H7B	0.0498	0.8668	0.5523	0.027*	
C8	0.2190 (2)	0.83140 (14)	0.45355 (7)	0.0219 (3)	
H8A	0.1415	0.9091	0.4239	0.026*	
H8B	0.3566	0.8720	0.4754	0.026*	
C8A	0.26523 (18)	0.70674 (13)	0.39857 (7)	0.0181 (3)	
H8A1	0.3744	0.6421	0.4257	0.022*	
C9	0.47226 (18)	0.72473 (13)	0.19025 (7)	0.0176 (3)	

### Atomic displacement parameters (Å^2^)

**Table:**

	*U*^11^	*U*^22^	*U*^33^	*U*^12^	*U*^13^	*U*^23^
O1	0.0244 (5)	0.0408 (6)	0.0233 (5)	0.0023 (4)	0.0086 (4)	−0.0059 (4)
C1	0.0194 (6)	0.0190 (6)	0.0190 (6)	−0.0020 (5)	0.0018 (5)	−0.0008 (5)
O2	0.0236 (5)	0.0198 (5)	0.0219 (4)	0.0021 (3)	0.0031 (3)	0.0030 (4)
C2	0.0171 (6)	0.0179 (6)	0.0181 (6)	0.0011 (4)	0.0028 (4)	0.0012 (5)
O3	0.0348 (5)	0.0249 (5)	0.0264 (5)	0.0084 (4)	0.0165 (4)	0.0053 (4)
C3	0.0206 (6)	0.0218 (6)	0.0176 (6)	−0.0016 (5)	0.0040 (5)	−0.0038 (5)
C4	0.0192 (6)	0.0197 (6)	0.0214 (6)	−0.0029 (5)	0.0046 (5)	−0.0027 (5)
C4A	0.0185 (6)	0.0173 (6)	0.0164 (6)	0.0023 (5)	0.0000 (4)	0.0026 (5)
C5	0.0180 (6)	0.0232 (6)	0.0182 (6)	0.0004 (5)	0.0007 (5)	−0.0003 (5)
C6	0.0227 (6)	0.0231 (7)	0.0175 (6)	0.0046 (5)	0.0029 (5)	0.0028 (5)
C7	0.0266 (7)	0.0237 (7)	0.0167 (6)	0.0021 (5)	0.0016 (5)	−0.0036 (5)
C8	0.0235 (6)	0.0220 (6)	0.0201 (6)	−0.0013 (5)	0.0016 (5)	−0.0028 (5)
C8A	0.0177 (6)	0.0189 (6)	0.0175 (6)	0.0009 (5)	0.0004 (5)	0.0005 (5)
C9	0.0155 (6)	0.0183 (6)	0.0190 (6)	−0.0012 (4)	0.0008 (4)	−0.0016 (5)

### Geometric parameters (Å, °)

**Table:**

O1—C6	1.2337 (15)	C4—H4A	0.9900
C1—C2	1.5319 (16)	C4—H4B	0.9900
C1—C8A	1.5373 (16)	C4A—C5	1.3443 (17)
C1—H1A	0.9900	C4A—C8A	1.5106 (16)
C1—H1B	0.9900	C5—C6	1.4597 (17)
O2—C9	1.2114 (15)	C5—H5	0.9500
C2—C9	1.5119 (16)	C6—C7	1.5006 (17)
C2—C3	1.5395 (16)	C7—C8	1.5290 (17)
C2—H2	1.0000	C7—H7A	0.9900
O3—C9	1.3315 (15)	C7—H7B	0.9900
O3—H3	0.888 (19)	C8—C8A	1.5287 (17)
C3—C4	1.5323 (16)	C8—H8A	0.9900
C3—H3A	0.9900	C8—H8B	0.9900
C3—H3B	0.9900	C8A—H8A1	1.0000
C4—C4A	1.5038 (17)		
			
C2—C1—C8A	113.85 (10)	C4A—C5—C6	122.56 (11)
C2—C1—H1A	108.8	C4A—C5—H5	118.7
C8A—C1—H1A	108.8	C6—C5—H5	118.7
C2—C1—H1B	108.8	O1—C6—C5	120.66 (11)
C8A—C1—H1B	108.8	O1—C6—C7	122.28 (11)
H1A—C1—H1B	107.7	C5—C6—C7	117.00 (10)
C9—C2—C1	110.17 (10)	C6—C7—C8	111.03 (10)
C9—C2—C3	108.79 (9)	C6—C7—H7A	109.4
C1—C2—C3	109.67 (9)	C8—C7—H7A	109.4
C9—C2—H2	109.4	C6—C7—H7B	109.4
C1—C2—H2	109.4	C8—C7—H7B	109.4
C3—C2—H2	109.4	H7A—C7—H7B	108.0
C9—O3—H3	110.2 (12)	C8A—C8—C7	111.88 (10)
C4—C3—C2	110.47 (9)	C8A—C8—H8A	109.2
C4—C3—H3A	109.6	C7—C8—H8A	109.2
C2—C3—H3A	109.6	C8A—C8—H8B	109.2
C4—C3—H3B	109.6	C7—C8—H8B	109.2
C2—C3—H3B	109.6	H8A—C8—H8B	107.9
H3A—C3—H3B	108.1	C4A—C8A—C8	111.88 (10)
C4A—C4—C3	112.89 (10)	C4A—C8A—C1	111.57 (9)
C4A—C4—H4A	109.0	C8—C8A—C1	109.47 (10)
C3—C4—H4A	109.0	C4A—C8A—H8A1	107.9
C4A—C4—H4B	109.0	C8—C8A—H8A1	107.9
C3—C4—H4B	109.0	C1—C8A—H8A1	107.9
H4A—C4—H4B	107.8	O2—C9—O3	122.57 (11)
C5—C4A—C4	121.19 (11)	O2—C9—C2	123.90 (11)
C5—C4A—C8A	122.59 (11)	O3—C9—C2	113.51 (10)
C4—C4A—C8A	116.14 (10)		
			
C8A—C1—C2—C9	175.69 (9)	C6—C7—C8—C8A	−55.49 (14)
C8A—C1—C2—C3	55.97 (13)	C5—C4A—C8A—C8	−15.56 (16)
C9—C2—C3—C4	−177.89 (10)	C4—C4A—C8A—C8	167.65 (10)
C1—C2—C3—C4	−57.33 (13)	C5—C4A—C8A—C1	−138.58 (12)
C2—C3—C4—C4A	53.90 (13)	C4—C4A—C8A—C1	44.63 (14)
C3—C4—C4A—C5	134.81 (12)	C7—C8—C8A—C4A	45.26 (13)
C3—C4—C4A—C8A	−48.36 (14)	C7—C8—C8A—C1	169.47 (10)
C4—C4A—C5—C6	171.69 (11)	C2—C1—C8A—C4A	−48.74 (13)
C8A—C4A—C5—C6	−4.94 (18)	C2—C1—C8A—C8	−173.13 (9)
C4A—C5—C6—O1	177.19 (12)	C1—C2—C9—O2	−37.96 (15)
C4A—C5—C6—C7	−5.68 (17)	C3—C2—C9—O2	82.29 (14)
O1—C6—C7—C8	−147.35 (12)	C1—C2—C9—O3	143.64 (10)
C5—C6—C7—C8	35.57 (15)	C3—C2—C9—O3	−96.11 (12)

### Hydrogen-bond geometry (Å, °)

**Table:**

*D*—H···*A*	*D*—H	H···*A*	*D*···*A*	*D*—H···*A*
O3—H3···O1^i^	0.888 (19)	1.79 (2)	2.6797 (13)	174.8 (17)
C2—H2···O2^ii^	1.00	2.40	3.3191 (15)	152
C7—H7A···O2^iii^	0.99	2.47	3.3708 (15)	151

Symmetry codes: (i) *x*+1, −*y*+3/2, *z*−1/2; (ii) −*x*+1, *y*−1/2, −*z*+1/2; (iii) *x*, −*y*+3/2, *z*+1/2.
